# Phenotypic Landscape of Pulmonary Neuroendocrine Tumors: Subtyped by OTP/ASCL1 Expression Correlated with Histology, Hormones and Outcome

**DOI:** 10.1007/s12022-025-09882-z

**Published:** 2025-11-06

**Authors:** Ayako Ura, Katja Evert, Matthias Evert, Bruno Märkl, Marcus Kremer, Elisa Moser, Hironobu Sasano, Yoshinori Okada, Katja Steiger, Carolin Mogler, Moritz Jesinghaus, Alexander von Werder, Seyer Safi, Hans Hoffmann, Günter Klöppel, Atsuko Kasajima

**Affiliations:** 1 Department of Pathology, TUM School of Medicine and Health, Technical University of Munich, Munich, Germany; 2 Institute of Pathology, University of Regensburg, Regensburg, Germany; 3 Institute of Pathology, Medical Faculty, University of Augsburg, Augsburg, Germany; 4 Institute of Pathology, Städtisches Klinikum München, Munich, Germany; 5 Department of Pathology, Tohoku University Graduate School of Medicine, Sendai, Japan; 6 Department of Thoracic Surgery, Tohoku University Graduate School of Medicine, Sendai, Japan; 7 Institute of Pathology, Philipps University of Marburg, Marburg, Germany; 8 Department of Internal Medicine II, TUM School of Medicine and Health, Technical University of Munich, Munich, Germany; 9 Division of Thoracic Surgery, TUM School of Medicine and Health, Technical University of Munich, Munich, Germany; 10 Bavarian Center for Cancer Research (BZKF), Munich, Germany

**Keywords:** Lung, NETs, Transcription factors, OTP, ASCL1, Histology, Hormones

## Abstract

Recent studies have shown that pulmonary neuroendocrine tumor (NET) subgroups, defined by the transcription factors OTP and ASCL1, correlate with age, sex, and tumor location. Their relationships with histology and hormone production, however, remain unclear. We analyzed 170 pulmonary NETs classified by OTP (O) and ASCL1 (A) expression into four groups: O + /A + , O + /A-, O-/A + , and O-/A-. Subgroups were assessed for histology, hormone expression, therapy-related markers, outcomes, and matched metastases. Among 152 resected primaries, O + /A + tumors (38%) were most frequent, occurring mainly in females (median age 72 years), and typically showed central or peripheral location, solid/spindle morphology with diffuse gastrin-releasing peptide (GRP), and focal ACTH/calcitonin. They also showed strong DLL3 expression and pronounced neuroendocrine cell hyperplasia. O + /A- tumors (23%) occurred predominantly in females (median age 56 years) with solid/trabecular patterns, occasional/ACTH, and strong SSTR2A/5 expression. O-/A- tumors (25%) were more common in males (median age 70 years), often central with solid/trabecular or oncocytic histology, serotonin expression (24%), and frequently SSTR2A-positivity. O-/A + tumors (14%) occurred across both sexes (median age 58 years), were centrally located, and solid, sometimes oncocytic features with moderate DLL3/SSTR2A expression. Metastases mirrored their primaries in transcription factor and hormone profiles. In the univariate analysis, OTP-negative tumors were associated with poorer disease-free survival (DFS). However, the multivariate analysis identified Ki67-based WHO grades (G1-G3) as the only independent prognostic factors. In conclusion, integrating OTP and ASCL1 refines pulmonary NET classification into four histologically and biologically distinct subgroups, providing additional insight into tumor heterogeneity. O + /A + tumors showed solid-spindle features and diffuse GRP and frequent ACTH expression, trabecular patterns characterized ASCL1-negative tumors, while oncocytic histology predominated in OTP-negative tumors, highlighting their role in defining tumor heterogeneity.

**Supplementary Information:**

The online version contains supplementary material available at 10.1007/s12022-025-09882-z.

## Introduction

Pulmonary neuroendocrine tumors (NETs), also known as pulmonary carcinoids, are well-differentiated neuroendocrine neoplasms that are classified as either typical carcinoid tumors (TCs) or atypical carcinoid tumors (ACs), depending on their mitotic activity and the presence of necrosis [1]. Recently, evidence for molecular and pathological diversity of pulmonary NETs has accumulated, indicating that pulmonary NETs exhibit a greater degree of heterogeneity than previously recognized [2]. Several studies have emphasized transcription factor-based subtyping in pulmonary NETs as a methodological approach offering a more refined classification framework [2–11]. In extrapulmonary organs, subtyping based on transcription factor expression have been reported in pituitary [12], pancreatic [13] and renal NETs [14]. This novel framework has been demonstrated to facilitate a more comprehensive understanding of the underlying tumor biology, thereby offering a more precise interpretation of tumor characteristics that may have significant implications for prognosis and therapeutic strategies.

The transcription factor orthopedia homeobox protein (OTP) has emerged as a highly specific and clinically relevant marker for pulmonary NETs. OTP is consistently expressed in TCs and in a subset of ACs, particularly those of peripheral origin [15–21], but is absent in small cell lung cancers (SCLCs), large cell neuroendocrine carcinomas (LCNECs), and non-pulmonary NETs [15, 22]. Clinically, nuclear OTP expression correlates with favorable prognosis, whereas its loss is associated with significantly poorer outcomes [7, 9, 10, 16, 21, 23]. Loss of OTP has also been linked to decreased CD44 expression, a cell adhesion molecule involved in tumor suppression and differentiation [16, 24]. Together, these findings underscore the diagnostic and prognostic utility of OTP in pulmonary NETs, adding value beyond conventional histopathological assessment.

Achaete-scute family bHLH transcription factor 1 (ASCL1), also known as MASH1, has been recognized since the late 1990 s for its essential role for neuroendocrine cell differentiation in the lung [25], central nervous system [26], and thyroid [27]. In lung tumors, ASCL1 is expressed in approximately 60% of SCLCs, 40% of LCNECs, and 50% of NETs [2, 7]. It has been demonstrated that combining ASCL1 with markers such as OTP, HNF1A or CD44 improves NETs subclassification, with identifying groups linked to clinical characteristics, including patient sex and tumor location [2, 7].

What remains unclear so far is the relationship between the molecular subgroups and the histological features of pulmonary NETs, particularly for OTP-positive and OTP-negative tumors [7, 17, 21]. Moreover, the connection between hormone expression, which is a component of the pulmonary NET phenotype [28], and transcription factor profiles, is unexplored.

This study has three main objectives. 1) to characterize the histological and hormonal profiles of resected primary pulmonary NETs across transcription factor-defined subgroups; 2) to assess intertumoral heterogeneity within these subgroups by analyzing matched metastatic tissues from the same patients; 3) to compare the histological and hormonal expression patterns of metastatic lesions with those of primary pulmonary NETs.

## Materials and Methods

### Tissue and Data Assembling

The supplementary Fig. 1 summarizes the procedure of our three-stage study on tissue samples from 170 clinically known patients with pulmonary NET. Of these patients, 152 underwent primary tumor resection (see Supplementary Table 1), which was performed at 13 different institutions in Germany (100 patients) and Japan (52 patients) between 2000 and 2024 (see Supplementary Table 2). In the first step, the 152 primary resected pulmonary NETs were examined for expression of the transcription factors OTP and ASCL1. In the next step, the intertumoral heterogeneity of the expression profiles of OTP and ASCL1 was investigated in 27 tumor samples from 12 patients, including 3 patients with primary tumors and synchronous nodal metastases, as well as 9 unoperated patients with multiple metastases available. Finally, the clinicopathological features of the 152 primary pulmonary NET patients were compared with those of 21 patients with metastatic pulmonary NETs (see Supplementary Fig. 1).

Diffuse idiopathic pulmonary neuroendocrine cell hyperplasia (DIPNECH) is defined by characteristic radiologic findings, including ventilatory abnormalities with mosaic attenuation or air trapping on imaging. All 10 clinically suspected DIPNECH cases were histologically confirmed by the presence of multifocal proliferation of pulmonary neuroendocrine cells within the bronchiolar epithelium. This was typically accompanied by fibrosis and tumorlets and was not associated with alternative etiologies. Three patients had Cushing syndrome. None was affected by multiple endocrine neoplasia type 1 (MEN1). In 140 patients with primary tumors, survival data (mean overall survival of 61 months) were obtained from the Bavarian Cancer Registry and/or the patients’ hospital records. Survival analysis was performed in 125 patients to evaluate disease-free survival (DFS) defined as the length of time in months after surgery which a patient remains alive without any evidence of recurrence or progression and disease-specific survival (DSS) defined as the time after surgery during which a patient has not died from the disease. Patients with an R1 resection and those with less than one month of follow-up (n = 14) were excluded. Local ethics committees at the Technical University of Munich, Germany (2022–396-S-DFG-SR) and Tohoku University in Sendai, Japan (2018–1–515) approved this study.

### Histological Evaluation

Three pathologists (A.U., G.K., A.K.) reviewed histological diagnoses based on H&E and immunostainings for cytokeratin 18, chromogranin A, synaptophysin, Ki-67, p53, and Rb1 (Supplementary Table 3). Tumors were classified as TC or AC according to the WHO thoracic neuroendocrine neoplasm criteria [1] and graded as NET G1, G2, or G3 per the WHO endocrine organ system [29]. According to predominant architecture, tumors were evaluated on whole slides sections and grouped as solid or trabecular. Solid pattern was defined as sheets or nests of tumor cells, whereas trabecular pattern referred to cords (reticulated) or glandular structures. If more than one pattern was present, the dominant one was recorded. Two tumors with solid paraganglioma-like pattern were included in the solid group, and two with trabecular paraganglioma-like pattern in the trabecular group. A spindle cell feature was defined when ≥ 5% of tumor cells had a vertical-to-horizontal ratio of at least 2:1 [30, 31]. An oncocytic feature was defined when ≥ 20% of tumor cells had abundant eosinophilic cytoplasm, centrally located nuclei, and prominent nucleoli [32, 33]. Other rare cytological variants were documented — clear cell (n = 5), plasmacytic (n = 1), pigmented (n = 1) — and included in the analysis as non-spindle/non-oncocytic tumors. We also assessed spread through air spaces (STAS), defined as the presence of tumor cells (either as single cells, clusters, or nests) in air spaces beyond the main tumor [34]. Pulmonary neuroendocrine cell hyperplasia (PNECH) was recorded when aggregated tumor cells (> 5) were found within intrabronchial mucosa, either focal or multifocal [2]. Many multifocal lesions were radiologically classified as DIPNECH.

### Tissue microarray Construction

Tissue microarrays (TMA) consisting of 2 cores, each 1.5 mm in diameter, from 1 FFPE block per case were constructed using the TMA Grand Master (Sysmex/3DHistech). Cores were obtained from representative central and peripheral tumor areas selected by two pathologists (A.K. and A.U.).

### Immunohistochemical Staining and Evaluation

The following markers were examined using immunohistochemical staining: the transcription factors OTP, ASCL1, HNF1A, and TTF1; CD44; four hormones (gastrin-releasing peptide [GRP], adrenocorticotropic hormone [ACTH], calcitonin, and serotonin); three therapy related markers somatostatin receptor 2 A [SSTR2A], SSTR5, and delta-like ligand 3 [DLL3]. To identify sustentacular cells, SOX10 was used instead of S100, as it allows specific detection of sustentacular cells and avoids confusion with S100-positive tumor cells [11]. OTP, ASCL1, HNF1A, TTF1, CD44, SSTR2A, SSTR5 and DLL3 were stained on TMAs, whereas hormones and SOX10 were stained on whole tissue slides. The staining results of the transcription factors on the TMAs were validated using whole slides of 20 randomly selected tumors. As a staining reference, OTP staining was performed on 15 randomly selected small cell lung cancers (SCLCs). In addition, ASCL1 staining was also performed on 50 randomly selected SCLCs, and H-score of 36 ASCL1 positive cases was compared with that of NETs.

Two pathologists (A.U. and A.K.) evaluated the slides using previously described evaluation methods and cut-cuff values [2, 9, 13, 21, 35–39]. The staining pattern of the adjacent lung tissue was evaluated and documented. The detailed staining and evaluation methods (scoring methods, cut-off values) are summarized in Supplementary Table 3. In brief, for the Ki67 index assessment, hot spot areas were selected and at least 500 tumor cells were counted on printed image [39]. OTP and ASCL1 were assessed using the H-score (0 to 300). Positivity was defined as an H-score of ≥ 10 for OTP and ≥ 40 for ASCL1, as previously reported [2]. Hormones were considered positive when ≥ 1% of cells stained and further graded as score 1 + (single cell positivity 1–9%), score 2 + (focal positivity 10–49%), and score 3 + (diffuse positivity ≥ 50%) [13].

### Statistical Analyses

All statistical analyses were performed using JMP Pro version 18.1.1 (SAS Institute Inc., Cary, NC, USA). Transcription factor expression on TMA and whole slides was compared using logistic regression and receiver operating characteristic (ROC) analysis, with whole slides as the reference. Clinicopathological features of transcription factor-based subgroups were compared with Pearson’s chi-squared or Fisher’s exact test. The Kruskal–Wallis test assessed continuous variables or scores across multiple non-normally distributed groups, as determined by the Shapiro–Wilk test. DFS and DSS were analyzed using the Kaplan–Meier method with significance by log-rank test. Multivariate survival analysis used the proportional hazards model. A p-value < 0.05 was considered statistically significant.

### Data Visualization

Heatmap visualizations were generated using R (version 4.x) and RStudio (version 2025.05.0, Build 496; Posit PBC, Boston, USA). The appropriate packages, including pheatmap (CRAN) and/or Complex Heatmap (Bioconductor), were used for clustering and color scaling as needed.

## Results

### OTP and ASCL1 Expression in 152 Primary Pulmonary NETs

#### Grouping

**Fig. 1:**
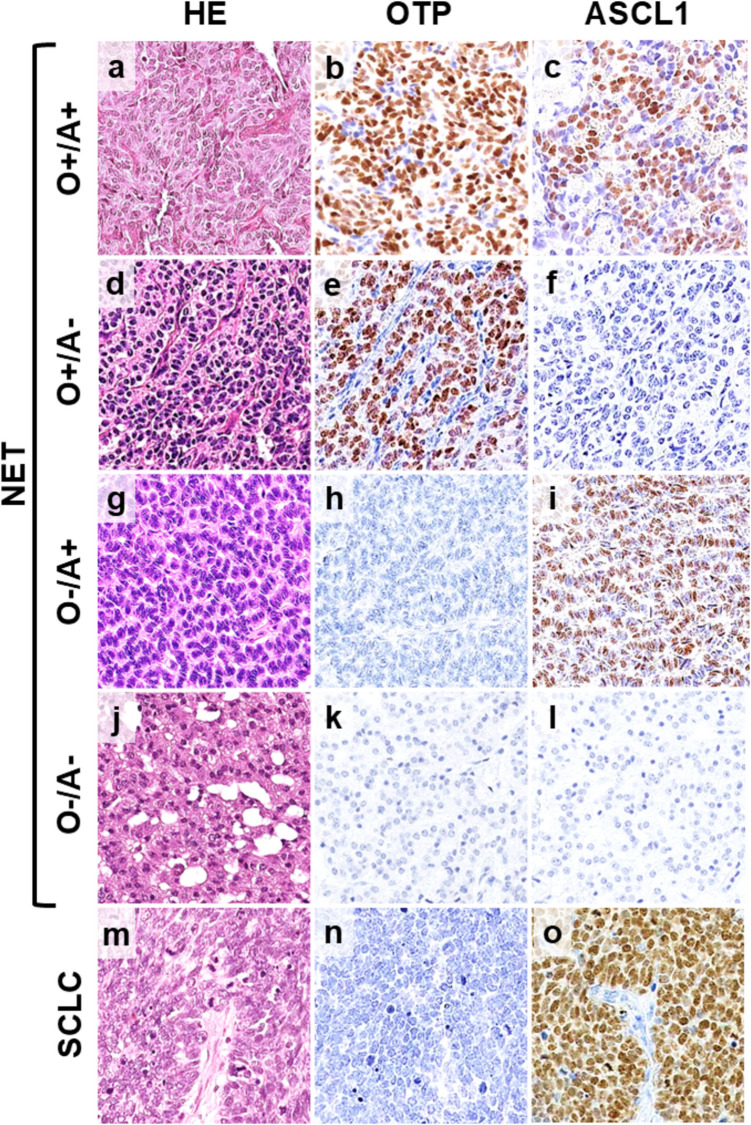
Histological features (**a**, **d**, **g**, **j**, and **m**) and immunohistochemical expression of OTP (**b**, **e**, **h**, **k**, and **n**) and ASCL1 (**c**, **f**, **i**, **l**, and **o**) in four subgroups of pulmonary neuroendocrine tumors (**a**-**l**) and small cell lung carcinoma (**m**–**o**). Abbreviations: NET, Neuroendocrine tumor; SCLC, Small cell lung carcinoma

**Fig. 2:**
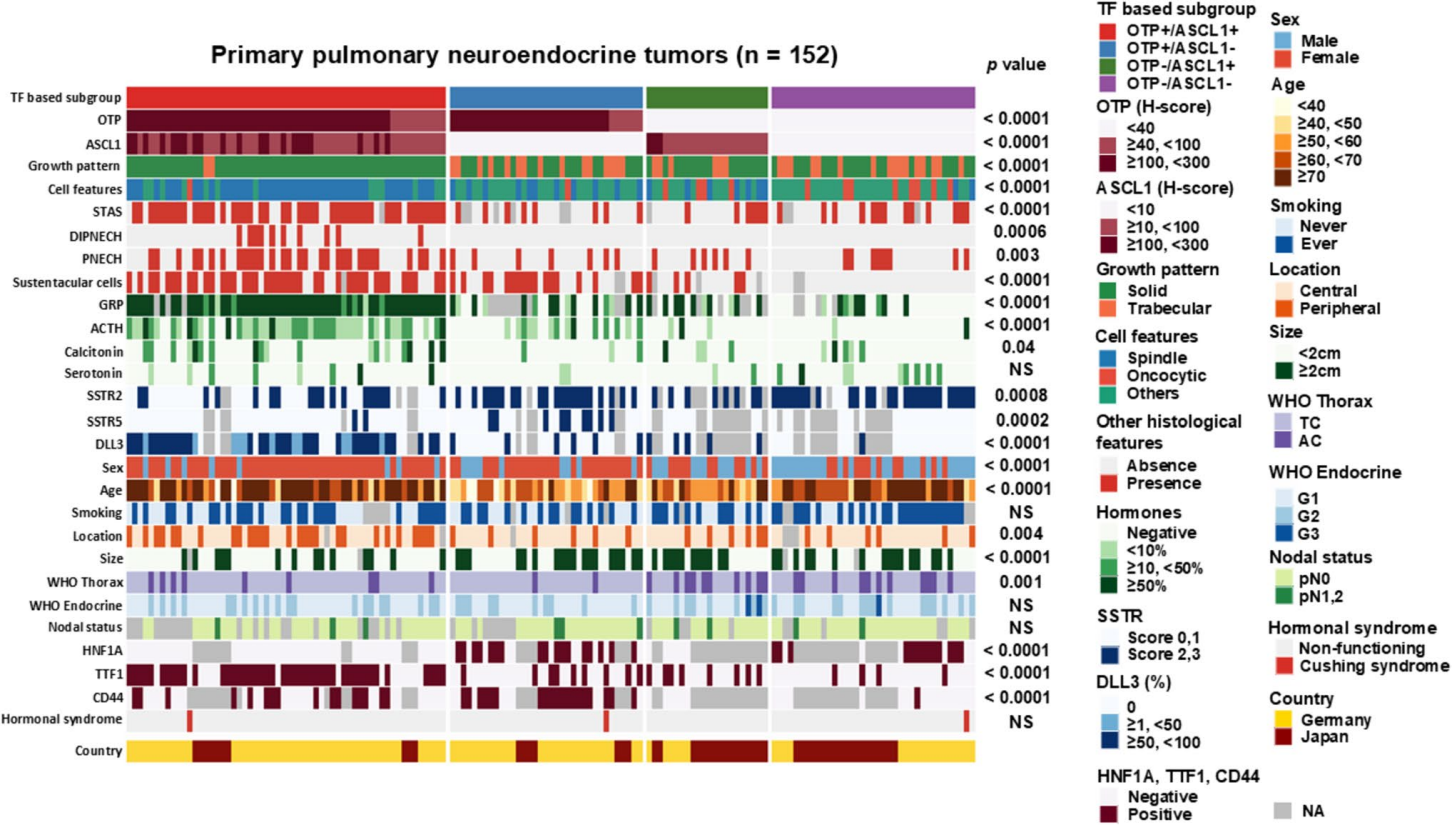
Heatmap overview of histological, immunohistochemical and clinical characteristics of 152 primary resected neuroendocrine tumor of the lung subgrouped by OTP and ASCL1 expression. Abbreviations: TF transcription factor, STAS spread through air spaces, DIPNECH diffuse idiopathic pulmonary neuroendocrine cell hyperplasia, PNECH pulmonary neuroendocrine cell hyperplasia, NE neuroendocrine, GRP gastrin-releasing peptide, ACTH adrenocorticotropic hormone, SSTR somatostatin receptor

OTP and ASCL1 expression were observed in 93 (61%) and 80 (47%) cases, respectively, with OTP expression being higher than ASCL1 expression (median H-scores of 110 and 10, respectively; see Fig. 1 and Supplementary Table 1**)**. OTP and ASCL1 expression were not observed in adjacent lung tissue. High concordance was observed between TMA and whole slide staining results for both OTP and ASCL1 (p < 0.001, area under the ROC curve (AUC) = 1.00 for both). None of the SCLC cases showed OTP expression (see Fig. 1). ASCL1 expression in NETs was lower than that in SCLCs (median H-score: 240, see Fig. 1). The tumors were grouped into four categories based on these expressions. The largest group had both OTP and ASCL1 (the O +/A + subgroup: 58/152, 38%). Those with only OTP (O +/A-) and those with neither marker (O-/A-) were similarly prevalent, with 35 (23%) and 37 (24%) tumors, respectively. The subgroup with only ASCL1 (O-/A +) was the least prevalent, with 22 cases (14%) (see Table 1). TTF1 expression was frequently observed in the O +/A + subgroup (74%), while HNF1A was expressed in the O +/A- (61%) and O-/A- (68%) subgroups (p < 0.05 for both). CD44 expression was predominantly observed in OTP-positive subgroups, particularly in the O +/A- subgroup (68%) (Table 1, Fig. 2).

**Table 1:** Comparison of clinicopathological features among four subtypes of lung neuroendocrine tumors based on expression of OTP and ASCL1

Characteristics		TF based subgroup				*p* value^A^	*p* value^B^
		I. O +/A + (OTP +/ASCL1 +)	II. O +/A- (OTP +/ASCL1-)	III. O-/A + (OTP-/ASCL1 +)	IV. A-/O- (OTP-/ASCL1-)		
Total (n)	152	58	35	22	37		
	(%)	38	23	14	24		
Age	Median (range)	72 (36–83)	56 (12–84)	58 (42–86)	70 (40–83)	< 0.0001^C^	< 0.001 I-II, I-III, II-IV, III-IV ^C^
Sex	Male	8 (14%)	9 (26%)	11 (50%)	25 (68%)	< 0.0001	< 0.001 I-III, I-IV, II-IV
	Female	50 (86%)	26 (74%)	11 (50%)	12 (32%)		
Smoking^a^	Never smoker	27 (51%)	17 (53%)	10 (48%)	9 (27%)	NS	0.03 I-IV, II-IV
	Smoker	26 (49%)	15 (47%)	11 (52%)	24 (73%)		
size (cm)^b^	Median (range)	1.2 (0.2–4.5)	2 (0.5–7.5)	1.9 (0.8–5)	1.8 (0.5–8.3)	< 0.0001^C^	< 0.001 I-II, I-III, I-IV ^C^
size (cm)	< 2 cm	42 (75%)	17 (49%)	11 (50%)	19 (54%)	0.04	0.01 I-II 0.04 I-IV
	≥ 2 cm	14 (25%)	18 (52%)	11 (50%)	16 (46%)		
Ki-67 (%)	Median (range)	2 (0.5–19)	2 (0.4–13.4)	2 (0.2–62)	2 (0.5–23.9)	NS^C^	NS^C^
Nodal status^c^	pN0	34 (92%)	24 (89%)	16 (89%)	29 (94%)	NS	NS
	pN1,2	3 (8%)	3 (11%)	2 (11%)	2 (6%)		
Location^d^	Central	29 (52%)	28 (82%)	16 (73%)	28 (82%)	0.004	0.004 I-II, I-IV
	Peripheral	27 (48%)	6 (18%)	6 (27%)	6 (18%)		
WHO	TC	49 (84%)	33 (94%)	12 (55%)	26 (70%)	0.001	< 0.001 II-III, II-IV
	AC	9 (16%)	2 (6%)	10 (45%)	11 (30%)		
Growth pattern	Trabecular	2 (3%)	15 (43%)	6 (27%)	16 (43%)	< 0.0001	< 0.001 I-II, I-III, I-IV
	Solid	56 (97%)	20 (57%)	16 (73%)	21 (57%)		
Cell features	Spindle	44 (76%)	11 (31%)	7 (32%)	2 (5%)	< 0.0001	< 0.001 I-II, I-III, I-IV 0.003 II-IV 0.02 III-IV
	Oncocytic	1 (2%)	1 (3%)	4 (18%)	8 (22%)		
	Others	13 (22%)	23 (66%)	11 (50%)	27 (73%)		
STAS^e^	Absence	13 (23%)	21 (72%)	15 (71%)	21 (62%)	< 0.0001	< 0.001 I-II, I-III, I-IV
	Presence	44 (77%)	8 (28%)	6 (29%)	13 (38%)		
DIPNECH	Absence	48 (83%)	35 (100%)	22 (100%)	37 (100%)	0.0006	0.01 I-II, I-IV 0.05 I-III
	Presence	10 (17%)	0	0	0		
PNECH	Absence	28 (48%)	28 (80%)	16 (72%)	29 (78%)	0.003	0.005 I-IV 0.006 I-II
	Presence	30 (52%)	7 (20%)	6 (27%)	8 (22%)		
Sustentacular cells^f^	Absence	18 (32%)	16 (48%)	14 (74%)	35 (100%)	< 0.0001	< 0.001 I-III, I-IV, II-IV 0.01 II-III, III-IV
	Presence	39 (68%)	17 (52%)	5 (26%)	0		
GRP^g^	Negative	0	11 (42%)	6 (38%)	24 (72%)	< 0.0001	< 0.001 I-II, I-III, I-IV 0.01 II-IV
	Positive	55 (100%)	15 (58%)	10 (62%)	9 (27%)		
ACTH	Negative	12 (21%)	21 (60%)	19 (86%)	35 (95%)	< 0.0001	< 0.001 I-II, I-III, I-IV, II-IV 0.02 III-IV 0.04 II-III
	Positive	46 (79%)	14 (40%)	3 (14%)	2 (5%)		
Calcitonin	Negative	40 (69%)	32 (91%)	18 (82%)	32 (86%)	0.04	0.01 I-II NS (0.052) I-IV
	Positive	18 (31%)	3 (9%)	4 (18%)	5 (14%)		
Serotonin	Negative	50 (86%)	33 (94%)	20 (91%)	28 (76%)	NS	0.03 II-IV
	Positive	8 (14%)	2 (6%)	2 (9%)	9 (24%)		
Functionality	Non-functioning	57 (98%)	24 (97%)	22 (100%)	36 (97%)	NS	NS
	Cushing syndrome	1 (2%)	1 (3%)	0	1 (3%)		
OTP H-score	Median (range)	210 (40–300)	240 (60–300)	0 (0)	0 (0)	< 0.0001 ^C^	< 0.001 I-III, I-IV, II-III, II-IV ^C^
ASCL1 H-score	Median (range)	65 (10–270)	0 (0–5)	50 (10–120)	0 (0–5)	< 0.0001 ^C^	< 0.001 I-II, I-IV, II-III, III-IV ^C^
TTF1	Negative	15 (26%)	26 (74%)	14 (64%)	33 (89%)	< 0.0001	< 0.001 I-II, I-IV 0.002 I-III
	Positive	43 (74%)	9 (26%)	8 (36%)	4 (11%)		
HNF1A^h^	Negative	46 (100%)	11 (39%)	6 (100%)	6 (32%)	< 0.0001	< 0.001 I-II, I-III 0.006 III-IV 0.01 II-III
	Positive	0	17 (61%)	0	13 (68%)		
CD44^i^	Negative	27 (63%)	9 (32%)	6 (100%)	18 (95%)	< 0.0001	< 0.001 I-IV 0.01 I-II
	Positive	16 (37%)	19 (68%)	0	1 (5%)		
SSTR2A^j^	Negative	35 (66%)	12 (36%)	6 (43%)	8 (24%)	0.0008	< 0.001 I-IV 0.01 I-II
	Positive	18 (34%)	21 (64%)	8 (57%)	26 (76%)		
SSTR5^k^	Negative	49 (96%)	21 (70%)	10 (100%)	23 (100%)	0.0002	< 0.001 I-II 0.007 II-IV
	Positive	2 (4%)	9 (30%)	0	0		
DLL3^l^	Negative	10 (19%)	24 (80%)	5 (50%)	23 (96%)	< 0.0001	< 0.001 I-II, I-IV 0.002 I-III
	Low expression	11 (21%)	0	0	0		
	High expression	31 (60%)	6 (20%)	5 (50%)	1 (4%)		

*TF*, transcription factor; *TC*, Typical carcinoid; *AC*, Atypical carcinoid; *DIPNECH*, Diffuse idiopathic pulmonary neuroendocrine cell hyperplasia; *PNECH*, Pulmonary neuroendocrine cell hyperplasia; *STAS*, Spread through air space; *NS*, Not significant. Footnote: Data missing in a) 13 (5 in I, 3 in II, 1 in III, 4 in IV), b) 4 (2 in I, 2 in IV), c) 39 (21 in I, 8 in II, 4 in III, 6 in IV), d) 6 (2 in I, 1 in II, 3 in IV), e) 11 (2 in I, 6 in II, 3 in IV), f) 8 (1 in I, 2 in II, 3 in III, 2 in IV), g) 22 (3 in I, 9 in II, 6 in III, 4 in IV), h) 53 (12 in I, 7 in II, 16 in III, 18 in IV), i) 56 (15 in I, 7 in II, 16 in III, 18 in IV), j) 18 (5 in I, 2 in II, 8 in III, 3 in IV), k) 38 (7 in I, 5 in II, 12 in III,14 in IV), l) 36 (6 in I, 5 in II, 12 in III, 13 in IV), A: Pearson’s chis-quare test among four subtypes, B: Fisher’s exact text between two subtypes, C: Kruskal–Wallis Test, DIPNECH: Defined by radiological and histological findings, PNECH: Defined by histological evidence of aggregated tumour cells (> 5) within the intrabronchial mucosa, either focal or multifocal, GRP: Refers to antisera directed against amphibian bombesin, which shares a homologous C-terminal structure with GRP

#### Clinicopathological Features

The O +/A + subgroup was characterized by older age (median 72 years), a high percentage of female patients (86%), smaller tumor size (median 1.2 cm), and peripheral or central location. The O +/A- subgroup was associated with a younger median age (56 years), female predominance (74%), larger tumor size (median 2 cm), and central location (82%). The O-/A + subgroup included relatively younger patients (median age 58 years) with an equal gender proportion, and centrally located tumors (73%). The O-/A- subgroup included older patients (median age 70 years), a higher proportion of males (68%), larger tumors (median 1.8 cm) and central location (82%). This subgroup showed the strongest association with smoking (73%) (Table 1, Fig. 2). There were no significant differences between the subgroups with respect to tumor laterality (right 56%, left 43%), Ki-67 index, or nodal status. The three patients with ectopic Cushing’s syndrome were distributed among the subgroups. The clinicopathologic characteristics of the four subgroups are detailed in Table 1 and summarized as a heat map in Fig. 2.

#### Histological Features

**Fig. 3:**
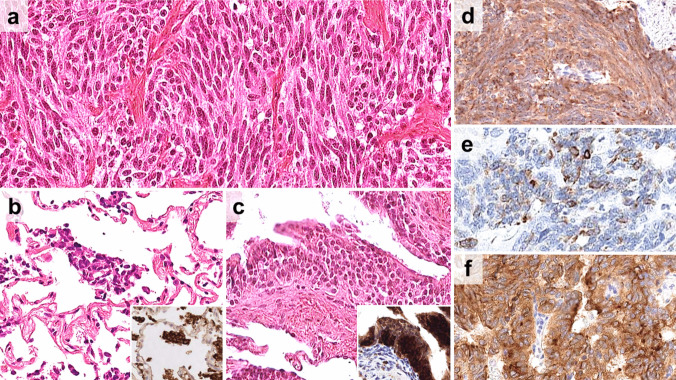
Histological (**a**, **b**, **c**) and immunohistochemical (**d**, **e**,** f**) features of pulmonary neuroendocrine tumor with OTP and ASCL1 expressions (the O +/A + subgroup). **a** Spindle shaped tumor cells showing a solid growth pattern, **b**) spread through air spaces (inset synaptophysin), and **c**) pulmonary neuroendocrine cell hyperplasia (inset Chromogranin A). **d** Strong and diffuse expression of gastrin-releasing peptide (GRP), **e**) single cell calcitonin expression and **f** diffuse strong membranous staining of DLL3

**Fig. 4:**
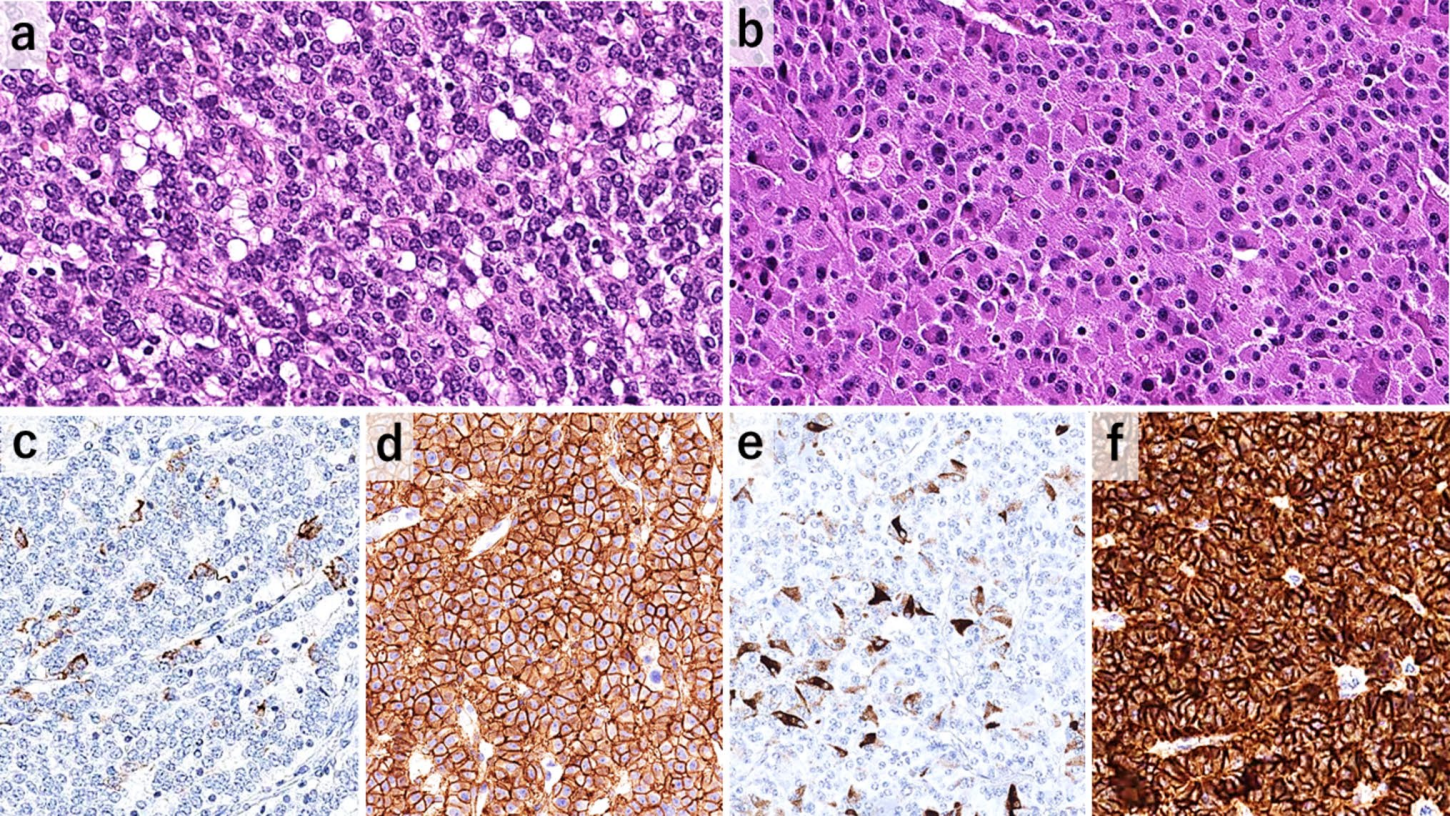
The histological (**a**, **b**) and immunohistochemical (**c**, **d**, **e**, **f**) images of the primary neuroendocrine tumors. Tumors with OTP positivity and ASCL negativity (the O +/A- subgroup) is characterized by **a**) a trabecular growth pattern, **c**) single cell expression for ACTH and **d**) diffuse strong membranous staining for SSTR5, while tumors without expression for OTP and ASCL (O-/A-) is associated with **b**) oncocytic cellular features, **e**) single cell serotonin expression and **f**) diffuse strong SSTR2A expression

A solid pattern was observed in 113/152 (74%) tumors, while a trabecular pattern in 39/152 (26%). Spindle cell and oncocytic features were present in 64/152 (42%) and 14/152 (9%), respectively. Spindle cell tumors were frequently associated with solid patterns (p < 0.0001). The O +/A + subgroup showed a solid growth pattern (97%) with spindle cell features (76%, Fig. 3). A solid growth pattern was also frequently observed in the O-/A + subgroup (73%), but with rare spindle cell features (32%). In contrast, the O +/A- and O-/A- subgroups showed less frequently a solid pattern (57% for both) in favor of a trabecular growth pattern (43% for both, Fig. 4 and Table 1). Spindle cell features were uncommon (31%) or rare (5%). Most of the oncocytic tumors were observed in either in the O-/A + (4/14) or in O-/A- (8/14) subgroups (Fig. 4, Table 1). PNECH were observed in 50/152 (33%) including all radiologically diagnosed DIPNECH cases. STAS and sustentacular cells were identified in 71/141 (50%) and 61/144 (42%) tumors, respectively. These findings were strongly associated with each other (p < 0.05) and predominantly found in the O +/A + subgroup (p < 0.05 for all, Fig. 2, Fig. 3b, c, Table 1).

#### Hormone Expression

Expression of GRP, ACTH, calcitonin, and serotonin was observed in 89/152 (68%), 65/152 (43%), 30/152 (20%), and 21/152 (14%) of the NETs, respectively (Supplementary Table 1). Most of the GRP expression was diffuse and strong, with a score of 3 + in 70/89 (79%) cases. In contrast, ACTH, calcitonin, and serotonin were mostly expressed in single cells or small cell clusters with a score of 1 + or 2 +. At least one hormone was expressed in 70% (107/152) of NETs (two hormones 28%, three hormones 13%, four hormones 3%). Tumor tissue from patients with Cushing’s syndrome showed diffuse and strong ACTH expression, in one case associated with single cell positivity for calcitonin and serotonin.

Expression of GRP, ACTH, and calcitonin was highly correlated (p < 0.05) and frequently observed in the O +/A + subgroup (100%, 79%, and 31%, respectively) (Fig. 2 and Fig. 3d, e). The O +/A- subgroup was associated with GRP (58%) and ACTH (40%) expression (Fig. 4c), but calcitonin expression was very rare (9%, Fig. 2). The O-/A + subgroup showed GRP expression (62%), but ACTH and calcitonin expression was rarely observed (14% and 18%, respectively) (Fig. 2, Table 1). The O-/A- subgroup had the highest frequency of serotonin expression (24%) (Fig. 4e).

No ACTH-, calcitonin-, or serotonin-positive cells were observed in the non-neoplastic lung tissue adjacent to the NETs, but GRP-positive cells were found scattered among chromogranin A-positive neuroendocrine cells in the bronchial mucosa (Supplementary Fig. 2).

#### Expression of Therapy-related Markers

SSTR2A and SSTR5 expressions were observed in 54% (73/134) and 10% (11/114) of the tumors, respectively. DLL3 expression was observed in 46% (54/116) of which 43/54 (80%) and 11/54 (20%) were defined as high and low expression, respectively. SSTR2A expression was most frequently observed in the O-/A- group (76%, 26/34, Fig. 4f), followed by the O +/A- (64%, 21/33) and O-/A + subgroups (57%, 8/14), and rarely in the O +/A + subgroup (34%, 18/53). Most of the SSTR5-expressing tumors were identified in the O +/A- subgroup (30%, 9/30, Fig. 4d). DLL3 was positive in 81% of the O +/A + tumors, mostly with high expression (74%, 31/42, Fig. 3f), and 50% of the O-/A + tumors expressed high DLL3 expression (Fig. 2, Table 1).

#### Patient Outcomes

**Fig. 5:**
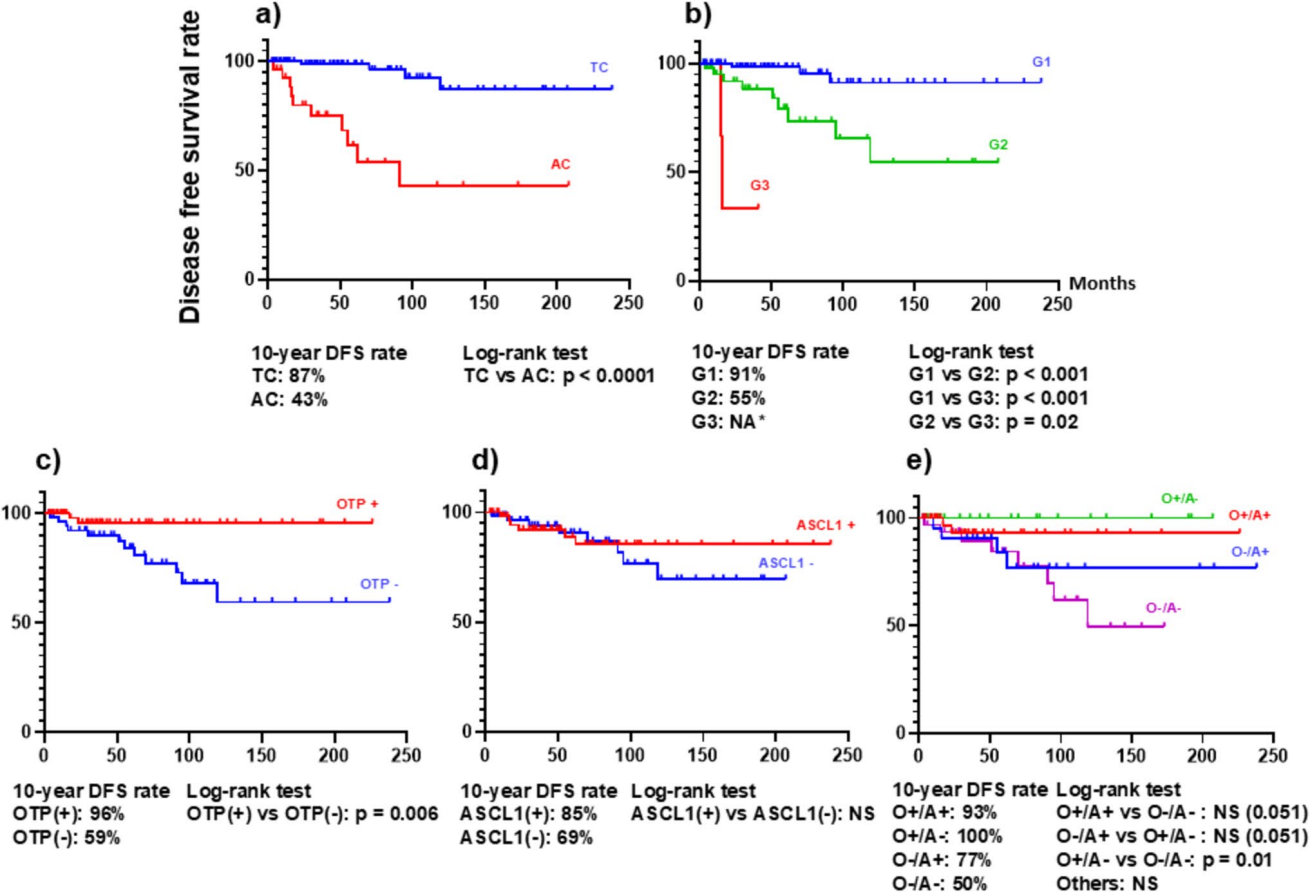
Kaplan–Meier curves for disease-free survival (DFS) of patients with primarily resected pulmonary neuroendocrine tumors, based on: **a**) the WHO classification for neuroendocrine neoplasms of the thoracic organs, **b**) the WHO classification for endocrine organs, **c**) OTP expression, **d**) ASCL1 expression, and **e**) the OTP/ASCL1-based subgroups

Regarding DFS, significant differences were observed in relation to the WHO classification (TC vs. AC, p < 0.0001, G1 vs. G2, p < 0.001, G1 vs. G3, p < 0.001, G2 vs. G3, p = 0.02) in univariate analysis. In addition, patients with OTP expression showed significantly better DFS compared to those with OTP-negativity (p = 0.006), whereas ASCL1 expression was not associated with DFS (Fig. 5). Among the four subgroups, the O +/A- subgroup demonstrated the most favorable outcome (10-year DFS: 100%), followed by O +/A + (93%), O-/A + (77%), and O-/A- (50%). A statistically significant difference was observed only between the O +/A- and O-/A- subgroups (p = 0.01), while no other pairwise comparisons reached significance (Fig. 5). Multivariate analysis revealed only Ki-67 based grading as independent prognostic factor for DFS (p = 0.04) (Supplementary Table 4). No significant difference in DSS was observed among the four subgroups (data not shown).

### Intertumoral Comparison of OTP and ASCL1 Expression in Tumors From 12 Patients

**Fig. 6:**
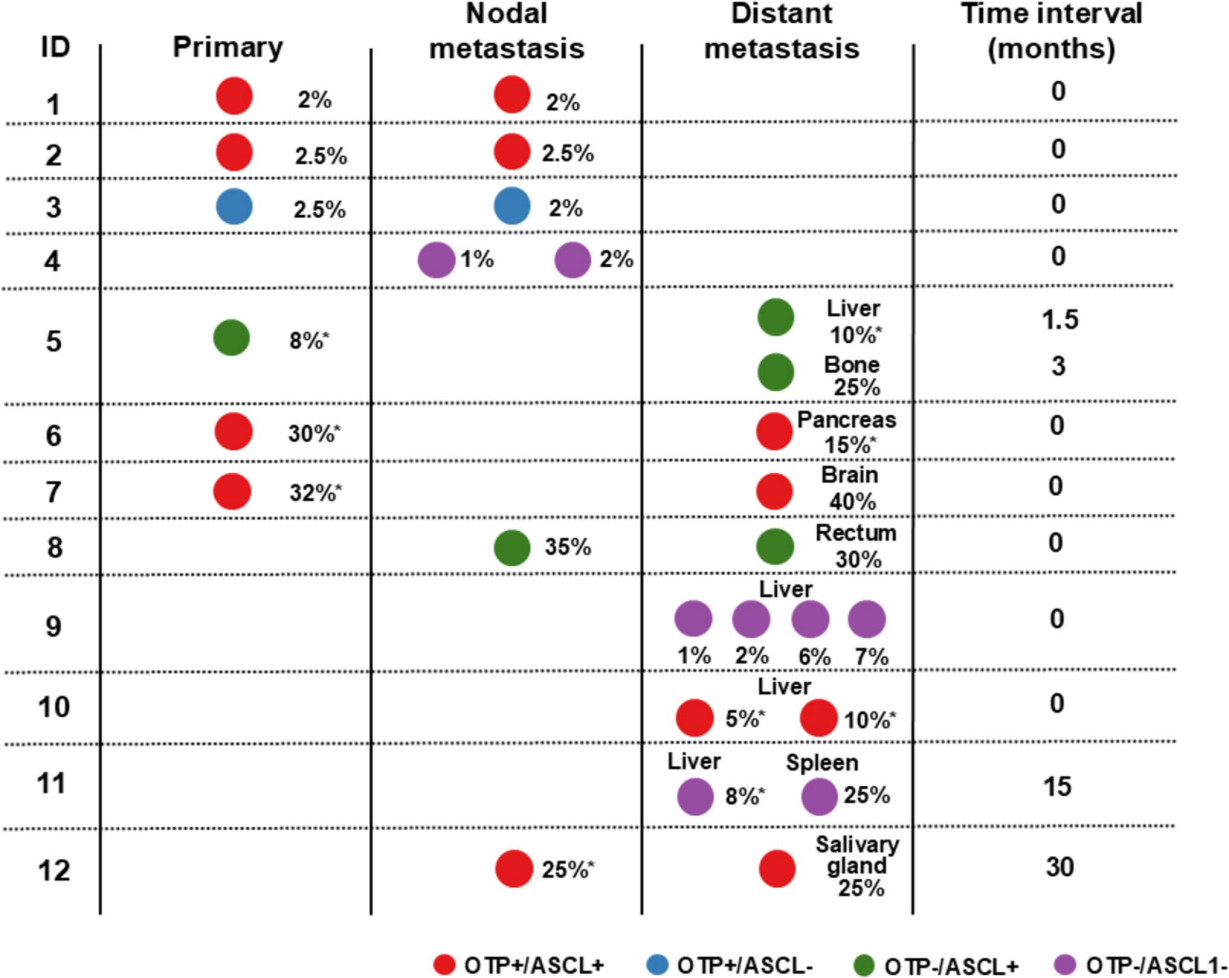
Pairwise comparison of pulmonary neuroendocrine tumor subgroups based on OTP and ASCL1 expression across multiple tumor samples from 12 patients. Percentage (%) indicates Ki-67 index of the tissues. An asterisk (*) indicates biopsy specimens

Concordant OTP and ASCL1 expression profiles were observed in all tumor tissues from each patient (Fig. 6). Six patients had O +/A + tumors, three patients had O-/A-, two patients had O-/A +, and one patient had O +/A-. Hormone expressions in metastatic tissue were consistent with that of the primary tumors and was also concordant among case-related metastases, except in two cases (ID 9 and 11) where focal serotonin expression was found in one of two metastases (see Supplementary Fig. 3).

### Comparison of Clinicopathologic Features of Primary and Metastatic Neuroendocrine Tumors

No differences in relative frequency of OTP and ASCL1 subgroups were found between primary and metastatic tumor tissues. A higher Ki-67 index (median: 9% vs. 2%, p < 0.0001) was observed in metastatic NETs than in primary NETs (Supplementary Table 5).

## Discussion

Our study of 152 primary pulmonary NETs showed that classification into four groups based on differential OTP and ASCL1 expression (O +/A +, O +/A-, O-/A +, O-/A-) revealed distinct clinicopathological, histological, hormonal features, and therapy-related marker expression.

**Fig. 7:**
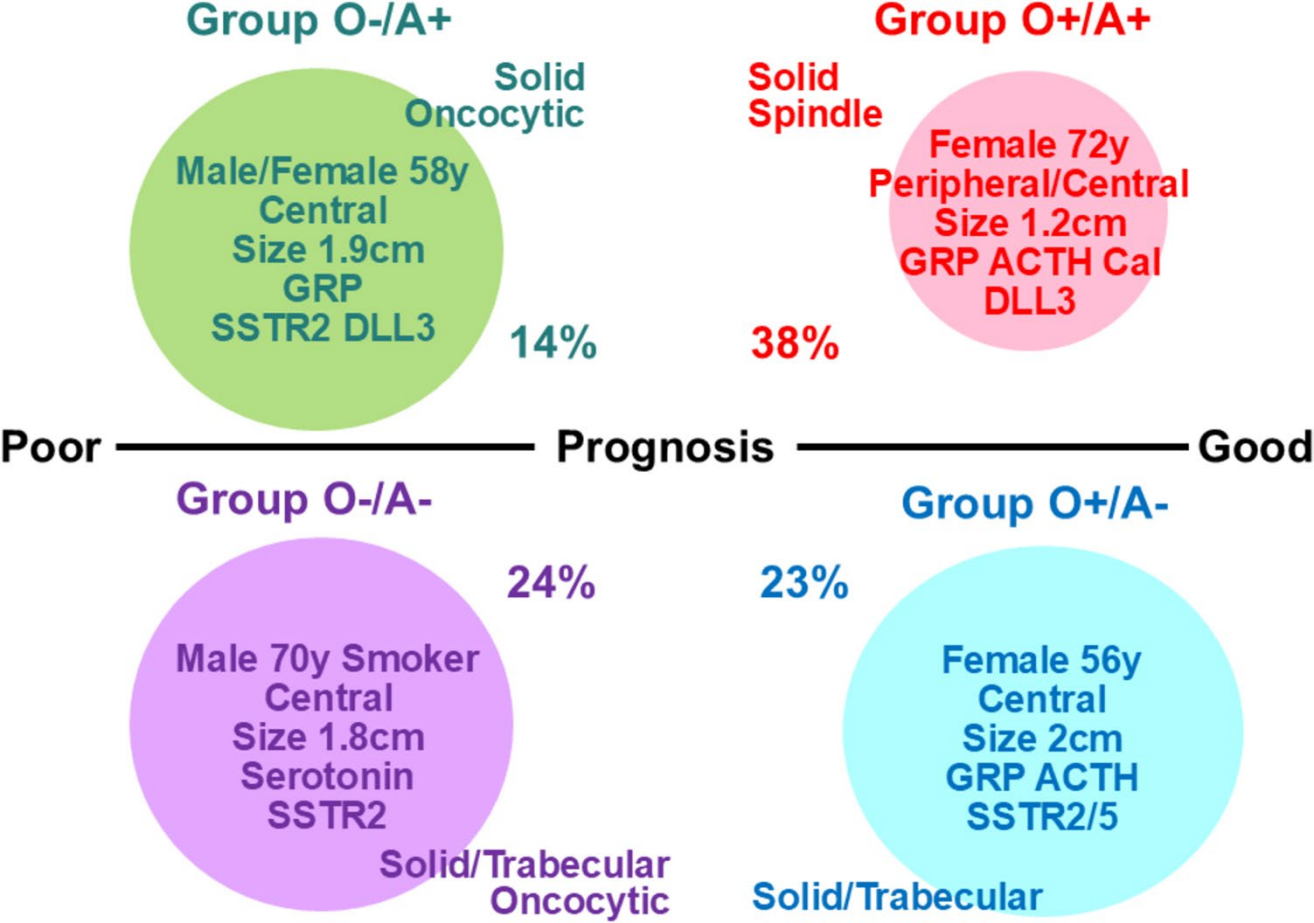
Clinicopathological and immunohistochemical features of four pulmonary neuroendocrine tumor subtypes classified by OTP (O) and ASCL1 (A) Expression. The predominant features, such as age and size, are given as medians. Abbreviations: GRP gastrin-releasing peptide, ACTH adrenocorticotropic hormone, Cal calcitonin

About two-thirds of cases occurred in females, distributed between the two OTP-positive subgroups. The O +/A + subgroup (38%) comprised mostly older women (median age, 72 years). Tumors were evenly central and peripheral, small (median 1.2 cm), typically showing a solid-spindle architecture, with diffuse GRP expression, frequent but focal ACTH, occasional calcitonin, abundant SOX10-positive sustentacular cells, and high DLL3 expression. They also had the highest incidence of PNECH, often multifocal, correlating with DIPNECH when radiologic data were available. In contrast, O +/A − tumors occurred in younger women (median 56 years), were predominantly central, showed a solid/trabecular architecture, had frequent GRP/ACTH but rare calcitonin/serotonin expression, and exhibited high SSTR2A/5 expression. The third (O-/A +, 14%) and fourth (O-/A-, 24%) pulmonary NET subgroups together comprised 38% of cases. O-/A + tumors occurred in patients with a median age of 58 years and an even gender distribution, were larger (1.9 cm), centrally located, with solid growth, occasional oncocytic features, frequent GRP, and enriched in therapy-related marker expressions. O-/A- tumors, typically in older male smokers (median age, 70 years), were large (1.8 cm), central, sometimes serotonin-positive with oncocytic features, and showed the highest SSTR2A but lacked DLL3 (Fig. 7). Prognostically, the OTP-negative subgroups, along with WHO histologic classification (AC) and Ki-67-based grading (G2, G3), were associated with shorter survival. On multivariate analysis, however, only Ki-67 grading remained an independent prognostic factor, consistent with prior reports [40–42].

The four tumor subgroups displayed notable differences in gender predominance, lung localization, tumor size, and smoking status. The available data do not allow firm explanations about the underlying basis of these associations. The only clear association was with smoking, which characterized the O-/A- subgroup—comprising mainly male smokers with tumors predominantly located in central lung regions.

To investigate intertumoral heterogeneity during progression, we analyzed transcription factor and hormone expression in matched primary and metastatic tissues. Histological and immunohistochemical features were also compared to identify progression-associated factors. Despite the small sample size, transcription factor and hormone profiles were stable between primary and metastatic tumors. The only difference was a significantly higher Ki-67 index in metastases, suggesting that increased proliferative activity is independent of transcription factor status and likely reflects molecular changes and/or treatment effects [43, 44].

Previous studies reported high OTP expression in most pulmonary NETs, with loss of nuclear OTP and/or low OTP mRNA correlating with reduced CD44 expression [16, 24]. OTP loss was more frequent in ACs than TCs [18, 21, 24] and strongly associated with poor prognosis [7, 16, 21, 45]. OTP is also a highly specific pulmonary NET marker, rarely expressed in other tumor types [15, 17, 18, 46]. These findings largely align with ours, though earlier studies reported lower frequencies of OTP-negative tumors. Notably, in our cohort, OTP-negative tumors were significantly more common among Japanese patients (35/52, 67%), while their clinicopathological features resembled those of German patients. The basis of this striking difference remains unclear and warrants further investigation.

Recent studies show that pulmonary NET classification can be refined by combining OTP expression with markers such as ASCL1 and HNF1A [2, 7]. Leunissen et al. analyzed a large cohort of resected primary pulmonary NETs using a transcription factor panel (OTP, ASCL1, HNF1A) and identified five molecular subgroups: two major OTP-positive (A1, A2) and three smaller OTP-negative (A1x, B, Bx). The A1 subgroup (OTP +/ASCL1 +/HNF1A-) included older women with predominantly peripheral tumors often associated with neuroendocrine hyperplasia. The A2 subgroup (OTP +/ASCL1-/HNF1A +) comprised younger patients, with balanced sex distribution and centrally located tumors. These clinicopathological profiles align with our findings in OTP-positive subgroups. OTP-negative subgroups showed male predominance and higher recurrence, partly overlapping with our O-/A + and O-/A- groups. Such parallels suggest biological relevance and reproducibility of transcription factor-based subtyping in pulmonary NETs [2]. Centonze et al. reported that ASCL1-positive/OTP-negative ACs had higher Ki-67 indices and poorer DFS. ASCL1 positivity increased recurrence risk 3.4-fold, and OTP loss independently predicted adverse outcomes, defining a high-risk group. By contrast, our study, including both TCs and ACs, found no prognostic value for ASCL1 alone and no significant Ki-67 differences across the four-transcription factor-based subgroups. The reasons for these discrepancies remain unclear, but differences in study populations—e.g., inclusion of only ACs in the Centonze cohort—may contribute [7].

The lack of differences in the Ki-67 index among the four subgroups is difficult to explain, but it likely reflects the small number of cases in each group and their widely varying Ki-67 values (see Ki-67 in Table 1), which obscure statistical significance. Additionally, the OTP/ASCL1 subgroups may capture transcriptional programs that influence outcomes independently of proliferation. This could explain why O-/A + and O-/A- tumors have a worse prognosis despite having similar median Ki-67 values.

This study provides a detailed analysis of histological and hormonal characteristics of pulmonary NET subgroups defined by OTP and ASCL1 expression. As reported previously [21, 30, 32], spindle cell features are common, often linked to peripheral location, sustentacular cells, OTP expression, and favorable outcomes. Spindle cells were strongly associated with solid growth and frequent in peripheral tumors, whereas trabecular growth predominated in central tumors. Other patterns, such as paraganglioma-like features, were rare (2%) and not subgroup-specific. Oncocytic pulmonary NETs, defined by large nuclei and prominent nucleoli [32, 33], share prognosis with non-oncocytic NETs and therefore must be distinguished from NEC [32, 33]. They were enriched in OTP-negative subgroups (O-/A + and O-/A-).

In 152 pulmonary NETs, GRP was the most frequently expressed hormone, followed by ACTH, calcitonin, and serotonin. GRP was identified in human lungs through immunostaining with antisera directed against amphibian bombesin, which share its C-terminal structure with that of GRP [47]. These anti-C-terminal sera were also used for the detection of GRP by radioimmunoassay [48]. GRP expression was also found in human lung NETs, most published between 1983 and 2006, but was not compared with histology, subtype, or clinical features [49–55]. In our series, GRP was common in solid spindle cell O +/A + tumors, evenly distributed between central and peripheral sites, often co-expressed with ACTH and occasionally calcitonin. GRP is a neuropeptide that stimulates gastric acid secretion and enhances gut motility. Despite its frequent detection in pulmonary NETs, no clinical syndrome attributable to GRP overproduction has been documented, even among 34 of 134 GRP-positive cases reported in the literature [49–55]. The absence of a GRP-related clinical syndrome is most likely explained by its very short plasma half-life (< 3 min) [56]. ACTH expression was detected in 43% of pulmonary NETs, typically with GRP and enriched in O +/A + NETs (see above). Three cases with diffuse ACTH expression were clinically associated with ectopic Cushing syndrome, whereas non-Cushing pulmonary NETs typically exhibited only focal, single-cell ACTH positivity [57, 58]. Calcitonin expression in pulmonary NETs was usually limited to single cells or small clusters. Only two prior studies examined calcitonin by immunohistochemistry, reporting positivity rates of 9–38% [49, 59], consistent with our findings (20%). In our series, calcitonin was most often seen in O +/A + tumors, frequently co-occurring with GRP and ACTH, and only rarely showing a diffuse pattern. Importantly, none of the calcitonin-positive patients exhibited clinical features of a calcitonin-related syndrome, such as diarrhea, fatigue, or weight loss [60].

Serotonin expression was found in 14% of tumors, most often in centrally located O-/A- cases. Positivity was usually limited to single cells or small clusters, and diffuse expression was rare. None of the serotonin-positive patients developed carcinoid syndrome, reported in ~ 7% of pulmonary NETs in recent literature [61]. Of the four hormones analyzed in our cohort of pulmonary NETs, only GRP is physiologically orthotopic to the adult lung and, accordingly, was also detected in non-neoplastic lung tissue adjacent to the resected tumors in our series. In contrast, the other hormones were absent from non-neoplastic lung tissues.

In conclusion, differential expression of OTP and ASCL1 allows for more precise pulmonary NET subtyping, identifying four distinct groups characterized by histology, hormone expression, and therapeutic markers. This transcription factor-based classification remains consistent throughout tumor progression and may enhance diagnostic accuracy and biological assessment. Although Ki-67 remains the strongest prognostic marker, loss of OTP expression is also associated with worse outcomes. This highlights the complementary value of OTP in risk stratification. Future investigations that integrate these phenotypic findings with genetic and epigenetic correlates may further clarify the biology of pulmonary NETs and inform clinical management.

## Supplementary Information

Below is the link to the electronic supplementary material.

ESM1 (DOCX 117 KB)

ESM2 (DOCX 2.51 MB)

ESM3 (DOCX 602 KB)

ESM4 (DOCX 21.1 KB)

ESM5 (DOCX 15.3 KB)

ESM6 (DOCX 22.3 KB)

ESM7 (DOCX 21.6 KB)

ESM8 (DOCX 19.9 KB)

## Data Availability

The Datasets used and analyzed during the current study are available from the corresponding author on reasonable request.
