# Supplementary material for: Phenotypic Landscape of Pulmonary Neuroendocrine Tumors: Subtyped by OTP/ASCL1 Expression Correlated with Histology, Hormones and Outcome
Source: Endocr Pathol. 2025 Nov 6;36(1):43. doi: 10.1007/s12022-025-09882-z (PMC12592246; doi:10.1007/s12022-025-09882-z)
Supplement: Supplementary file 1 — (DOCX 117 KB) [file 12022_2025_9882_MOESM1_ESM.docx]

Supplementary Figure 1: Workflow of the study. The cohort consists of 170 patients with pulmonary neuroendocrine tumor (NET) including 152 patients who underwent primary resection and 21 patients with metastatic and non-operated tumors. Multiple tumor tissues were available from 12 patients including 3 primary resected patients with synchronous nodal metastasis, and 9 non-operated patients with multiple metastatic tissues. Histopathological features of 152 primary resected NETs were compared with those of 21 metastatic pulmonary NETs.


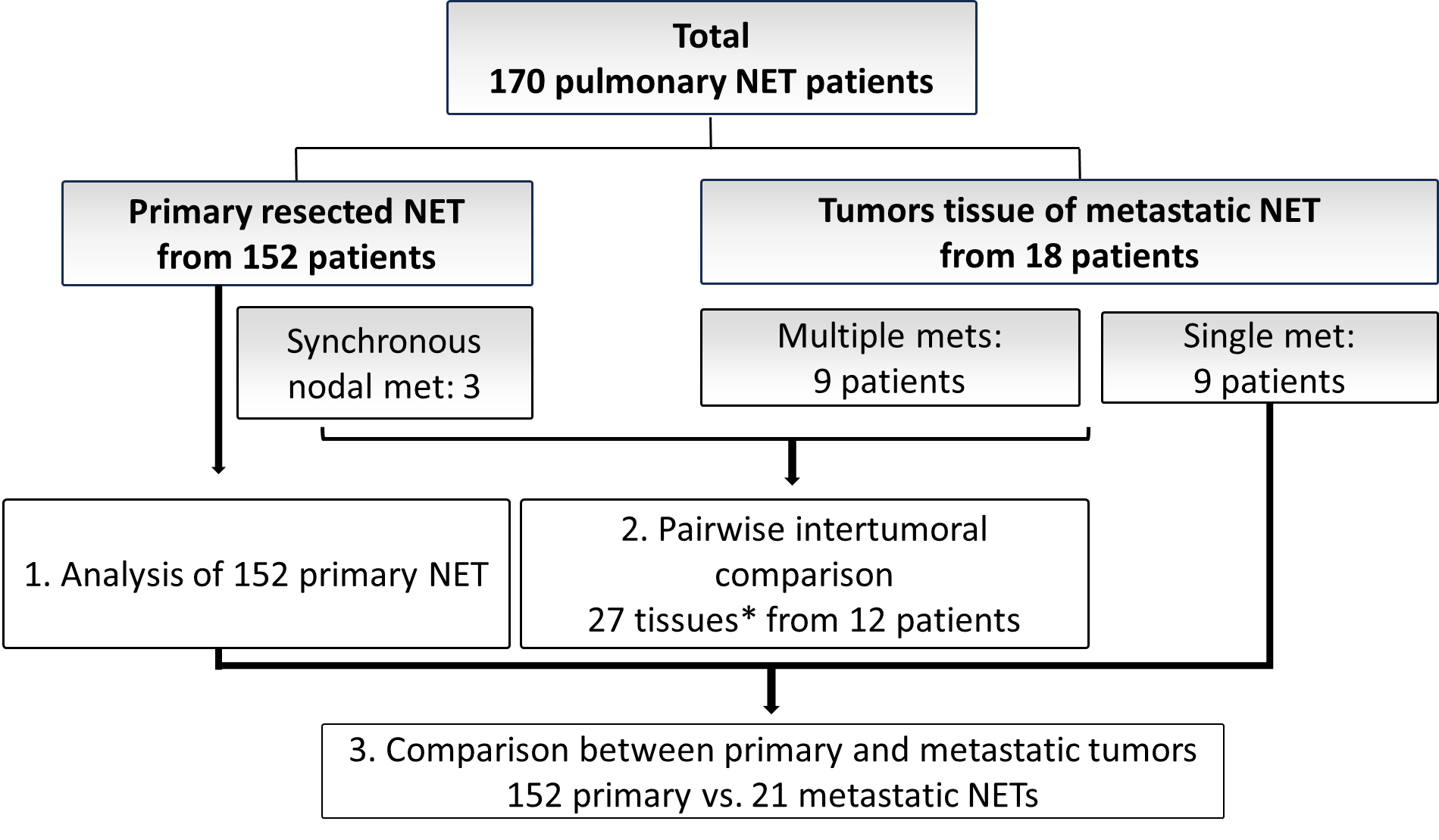


Footnote: *8 biopsy specimens and 19 resected tumor tissues.

Endocrine Pathology, A. Ura et al. Department of Pathology. Technical University Munich, TUM school of Medicine and Health, Munich, Germany, atsuko.kasajima@tum.de
