# Supplementary material for: Phenotypic Landscape of Pulmonary Neuroendocrine Tumors: Subtyped by OTP/ASCL1 Expression Correlated with Histology, Hormones and Outcome
Source: Endocr Pathol. 2025 Nov 6;36(1):43. doi: 10.1007/s12022-025-09882-z (PMC12592246; doi:10.1007/s12022-025-09882-z)
Supplement: Supplementary file 2 — (DOCX 2.51 MB) [file 12022_2025_9882_MOESM2_ESM.docx]

Supplementary Figure 2. Hormone expression in normal bronchial epithelium. Scattered chromogranin A–positive non-neoplastic neuroendocrine cells that are mostly positive for GRP (yellow arrows); in contrast, ACTH-, calcitonin-, and serotonin-positive cells are not identified.


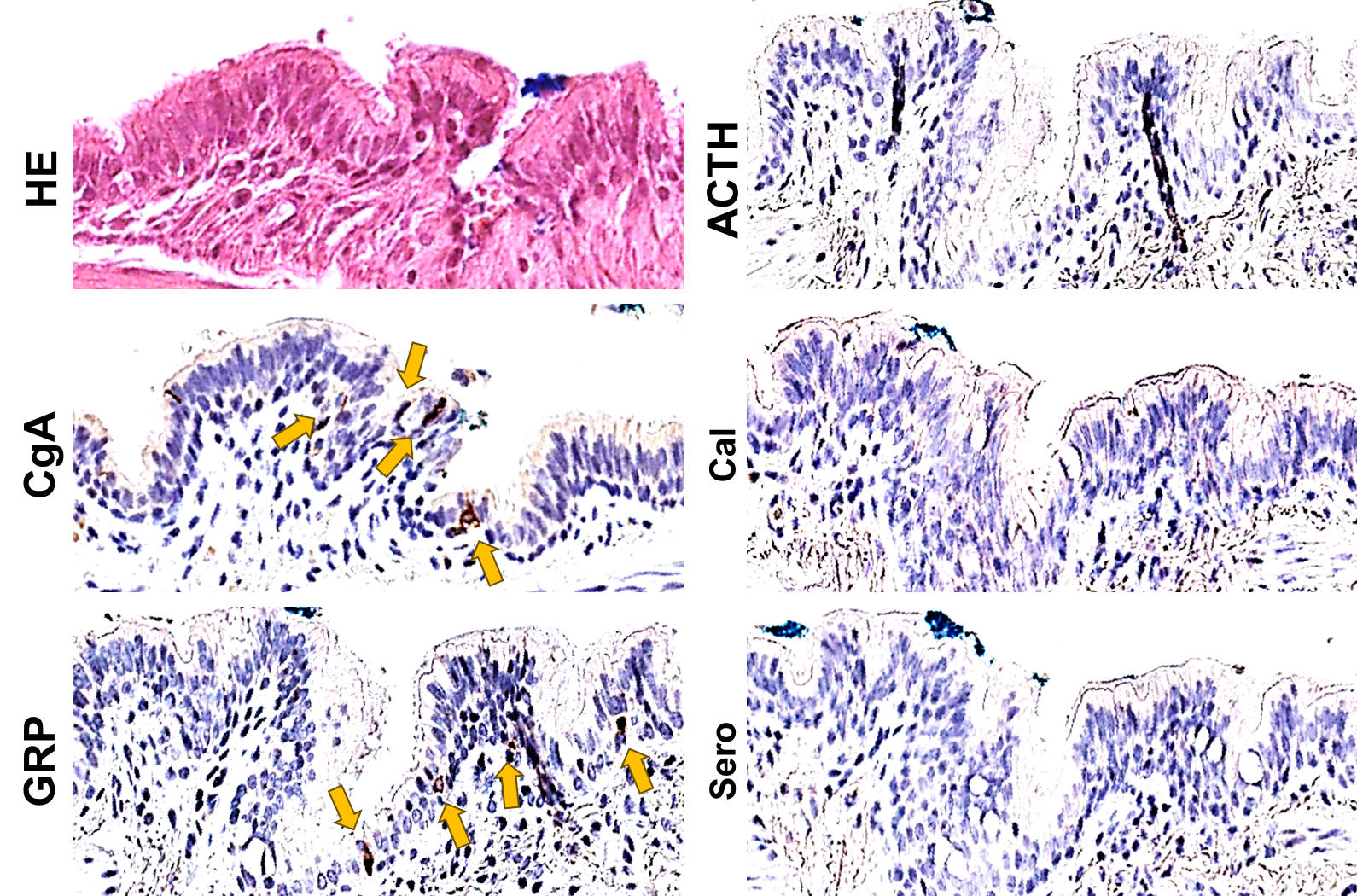


Abbreviations: CgA, Chromogranin A; GRP, Gastrin-releasing peptide; ACTH, Adrenocorticotropic hormone; Cal, Calcitonin; Sero, Serotonin.

Endocrine Pathology, A. Ura et al. Department of Pathology. Technical University Munich, TUM school of Medicine and Health, Munich, Germany, atsuko.kasajima@tum.de
