# Supplementary material for: Phenotypic Landscape of Pulmonary Neuroendocrine Tumors: Subtyped by OTP/ASCL1 Expression Correlated with Histology, Hormones and Outcome
Source: Endocr Pathol. 2025 Nov 6;36(1):43. doi: 10.1007/s12022-025-09882-z (PMC12592246; doi:10.1007/s12022-025-09882-z)
Supplement: Supplementary file 3 — (DOCX 602 KB) [file 12022_2025_9882_MOESM3_ESM.docx]

Supplementary Figure 3: Hormone expression profiles (GRP, ACTH, calcitonin, serotonin) across multiple tumor samples from the same patients. Profiles were concordant across tissues in 9 patients, whereas intertumoral heterogeneity was observed in cases 9 and 11 (serotonin IHC score 2 vs 0). Case 4 had ectopic Cushing syndrome.


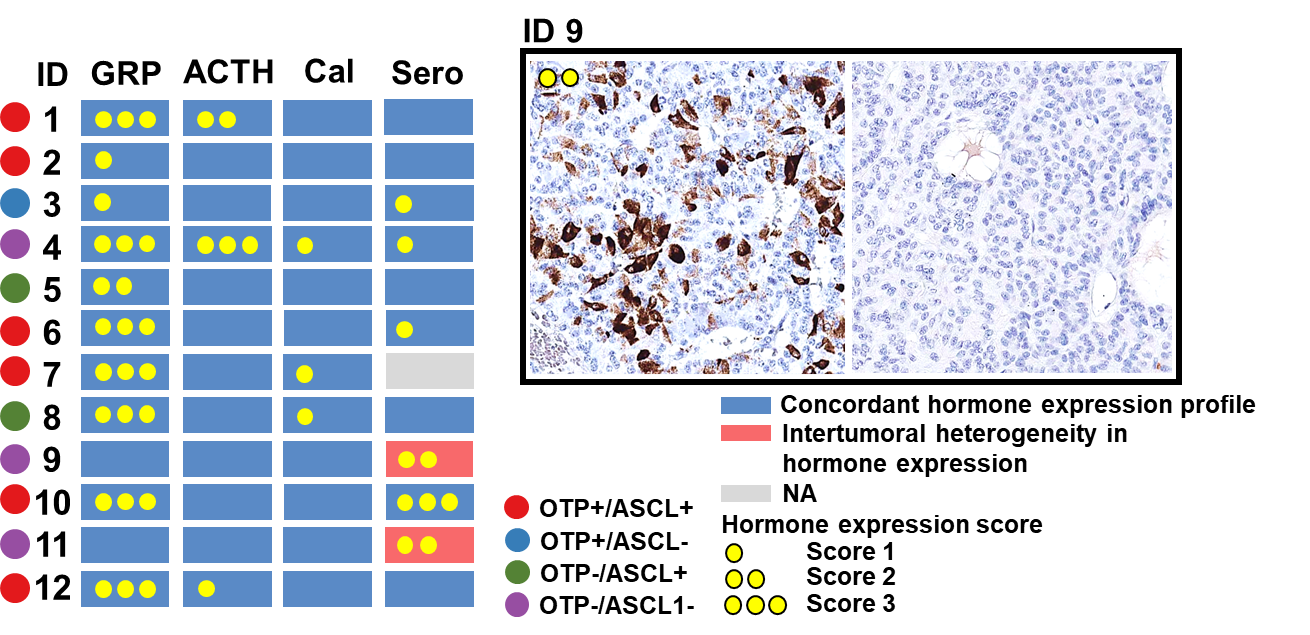


Abbreviations: GRP, Gastrin-releasing peptide; ACTH, Adrenocorticotropic hormone; Cal, Calcitonin; Sero, Serotonin; NA, not available.

Endocrine Pathology, A. Ura et al. Department of Pathology. Technical University Munich, TUM school of Medicine and Health, Munich, Germany, atsuko.kasajima@tum.de
