# Supplementary material for: Phenotypic Landscape of Pulmonary Neuroendocrine Tumors: Subtyped by OTP/ASCL1 Expression Correlated with Histology, Hormones and Outcome
Source: Endocr Pathol. 2025 Nov 6;36(1):43. doi: 10.1007/s12022-025-09882-z (PMC12592246; doi:10.1007/s12022-025-09882-z)
Supplement: Supplementary file 4 — (DOCX 21.1 KB) [file 12022_2025_9882_MOESM4_ESM.docx]

Supplementary Table 1: Clinicopathological features of 152 patients with primary resected pulmonary neuroendocrine tumors.

|  | Total | 152 | (%) |
| --- | --- | --- | --- |
| Age | Median (range) | 67 (12-86) |  |
| Sex | Male | 53 | 35 |
|  | Female | 99 | 65 |
| Smoking^a^ | Never smoker | 63 | 45 |
|  | Smoker | 76 | 55 |
| Size (cm)^b^ | Median (range) | 1.6 (0.2-8.3) |  |
| Ki-67 (%) | Median (range) | 2 (0.2-62) |  |
| WHO Thorax | TC | 120 | 79 |
|  | AC | 32 | 21 |
| WHO Endocrine | G1 | 97 | 64 |
|  | G2 | 52 | 34 |
|  | G3 | 3 | 2 |
| pT^c^ | pT1/2 | 117 | 95 |
|  | pT3/4 | 6 | 5 |
| Nodal status^d^ | pN0 | 103 | 91 |
|  | pN1/2 | 10 | 9 |
| Location^e^ | Central | 101 | 69 |
|  | Peripheral | 45 | 31 |
| Functionality | Non functioning | 149 | 98 |
|  | Cushing syndrome | 3 | 2 |
| Growth pattern | Solid | 113 | 74 |
|  | Trabecular | 39 | 26 |
| Spindle cells | No | 88 | 58 |
|  | Yes | 64 | 42 |
| Oncocytic cells | No | 138 | 91 |
|  | Yes | 14 | 9 |
| STAS^f^ | Absence | 70 | 50 |
|  | Presence | 71 | 50 |
| DIPNECH | Absence | 142 | 93 |
|  | Presence | 10 | 7 |
| PNECH | Absence | 101 | 66 |
|  | Presence | 51 | 34 |
| Sustentacular cells^g^ | Absence | 83 | 58 |
|  | Presence | 61 | 42 |
| GRP^h^ | Negative | 41 | 31 |
|  | Positive | 89 | 68 |
| ACTH | Negative | 87 | 57 |
|  | Positive | 65 | 43 |
| Calcitonin | Negative | 122 | 80 |
|  | Positive | 30 | 20 |
| Serotonin | Negative | 131 | 86 |
|  | Positive | 21 | 14 |
| OTP | Negative | 59 | 39 |
|  | Positive | 93 | 61 |
| OTP H-score | Median (range) | 110 (0-300) |  |
| ASCL1 | Negative | 72 | 53 |
|  | Positive | 80 | 47 |
| ASCL1 H-score | Median (range) | 10 (0-270) |  |
| TTF-1 | Negative | 88 | 42 |
|  | Positive | 64 | 58 |
| HNF1A^i^ | Negative | 69 | 70 |
|  | Positive | 30 | 30 |
| CD44^j^ | Negative | 60 | 63 |
|  | Positive | 36 | 37 |
| SSTR2A^k^ | Negative | 61 | 46 |
|  | Positive | 73 | 54 |
| SSTR5^l^ | Negative | 103 | 90 |
|  | Positive | 11 | 10 |
| DLL3^m^ | Negative | 62 | 53 |
|  | Low expression | 11 | 9 |
|  | High expression | 43 | 37 |

Abbreviations: STAS, spread through air spaces; DIPNECH, diffuse idiopathic pulmonary neuroendocrine cell hyperplasia; PNECH, pulmonary neuroendocrine cell hyperplasia; TC, typical carcinoid; AC, atypical carcinoid.

Footnote: Data missing in: a)13; b)4; c)29; d)39; e)6; f)11; g)8; h)22, i)53, j)56, k)18, l)38, m)36 cases, DIPNECH: Defined by radiological and histological findings, PNECH: Defined by histological evidence of aggregated tumour cells (>5) within the intrabronchial mucosa, either focal or multifocal, GRP: Refers to antisera directed against amphibian bombesin, which shares a homologous C-terminal structure with GRP.
