# Supplementary material for: Phenotypic Landscape of Pulmonary Neuroendocrine Tumors: Subtyped by OTP/ASCL1 Expression Correlated with Histology, Hormones and Outcome
Source: Endocr Pathol. 2025 Nov 6;36(1):43. doi: 10.1007/s12022-025-09882-z (PMC12592246; doi:10.1007/s12022-025-09882-z)
Supplement: Supplementary file 5 — (DOCX 15.3 KB) [file 12022_2025_9882_MOESM5_ESM.docx]

Supplementary Table 2: Number of patients with primary resected pulmonary neuroendocrine tumors by institution.

|  | Institution | Country | n |
| --- | --- | --- | --- |
| 1 | University Hospital rechts der Isar, Technical University Munich | Germany | 66 |
| 2 | University Hospital Regensburg | Germany | 22 |
| 3 | University Hospital Augsburg | Germany | 7 |
| 4 | Städtisches Klinikum München | Germany | 5 |
| 5 | Aomori Prefectural Central Hospital | Japan | 7 |
| 6 | Ishinomaki Red Cross Hospital | Japan | 4 |
| 7 | Iwate Prefectural Central Hospital | Japan | 10 |
| 8 | Iwate Prefectural Isawa Hospital | Japan | 3 |
| 9 | Miyagi Cancer Center | Japan | 8 |
| 10 | Miyagi Cardiovascular and Respiratory Center | Japan | 3 |
| 11 | Sendai Medical Center | Japan | 4 |
| 12 | Tohoku Medical Pharmaceutical University | Japan | 1 |
| 13 | Tohoku University Hospital | Japan | 12 |
| Total |  |  | 152 |
