# Supplementary material for: Phenotypic Landscape of Pulmonary Neuroendocrine Tumors: Subtyped by OTP/ASCL1 Expression Correlated with Histology, Hormones and Outcome
Source: Endocr Pathol. 2025 Nov 6;36(1):43. doi: 10.1007/s12022-025-09882-z (PMC12592246; doi:10.1007/s12022-025-09882-z)
Supplement: Supplementary file 6 — (DOCX 22.3 KB) [file 12022_2025_9882_MOESM6_ESM.docx]

Supplementary Table 3. Summary of detailed information of immunohistochemical staining and evaluation methods

| Antibody | Source | Clone/ Catalog number | Dilution | Automated platform | Product description | Section types | Staining pattern | Evaluation/ Scoring methods | Reference number |
| --- | --- | --- | --- | --- | --- | --- | --- | --- | --- |
| Synaptophysin | Invitrogen, Carlsbad, USA | SP11 | 1:50 | Leica^a^ | Rabbit, monoclonal | WS | Cytoplasmic | Either focal or diffuse staining was regarded as positive | - |
| Chromogranin A | Abcam, Cambridge, UK | ab15160 | 1:300 | Leica^a^ | Rabbit, polyclonal | WS | Cytoplasmic | Either focal or diffuse staining was regarded as positive | - |
| CK18 | Sigma-Aldrich Chemie GmbH, Taufkirchen, Germany | CY-90 | 1:2000 | Ventana^b^ | Mouse, monoclonal | WS | Cytoplasmic | Either focal or diffuse staining was regarded as positive | - |
| Ki-67 | Dako, Glostrup, Denmark | MIB-1 | 1:50 | Ventana^b^ | Mouse, monoclonal | WS | Nuclear | Ki-67 index (% in hot spot, >500 cells) | [39] |
| p53 | Dako | DO-7 | 1:200 | Ventana^b^ | Mouse, monoclonal | TMA | Nuclear | Normal if <20% of tumor nuclei positive,  Abnormal if complete loss expression or ≥20% strong nuclear positivity | [39] |
| Rb1 | BD Biosciences, Franklin Lakes, NJ, USA | G3-245 | 1:100 | Ventana^b^ | Mouse, monoclonal | TMA | Nuclear | Normal if nuclear staining in 10% or more tumor cells,  Abnormal if loss expression in >90% of tumor nuclei | [39] |
| TTF-1 | Zytomed Systems, Berlin, Germany | 8G7G3/1 | 1:50 | Leica^a^ | Mouse, monoclonal | TMA | Nuclear | Negative H-Score < 5, Positive H-score 5 or higher | [35] |
| ASCL1 | BD Biosciences | 24B72D11.1 | 1:100 | Leica^a^ | Mouse, monoclonal | TMA | Nuclear | Negative H-Score < 10, Positive H-score 10 or higher | [2] |
| OTP | Invitrogen | CL11225 | 1:50 | Ventana^b^ | Mouse, monoclonal | TMA | Nuclear | Negative H-Score < 40, Positive H-score 40 or higher | [2] |
| HNF1A | Invitrogen | GT4110 | 1:300 | Leica^a^ | Rabbit, monoclonal | TMA | Nuclear | Negative H-Score < 30, Positive H-score 30 or higher | [2] |
| CD44 | Agilent, Santa Clara, USA | DF1485 | 1:50 | Ventana^b^ | Mouse, monoclonal | WS | Membranous/ Cytoplasmic | Negative H-Score < 30, Positive H-score 30 or higher | [9] |
| ACTH | Dako | 02A3 | 1:2000 | Ventana^b^ | Mouse, monoclonal | WS | Cytoplasmic | Negative: Score 0 (no positive cells),  Positive: Score 1 (1-9%), Score 2 (10%-49%), Score 3 (≥50%) | [13] |
| Calcitonin | Leica Biosystems, Nußloch, Germany | CL1948 | 1:600 | Ventana^b^ | Mouse, monoclonal | WS | Cytoplasmic | Negative: Score 0 (no positive cells),  Positive: Score 1 (1-9%), Score 2 (10%-49%), Score 3 (≥50%) | [13] |
| GRP/bombesin^c^ | ImmunoStar, Hudson, WI, USA | IRCP069 | 1:300 | Ventana^b^ | Rabbit, polyclonal | WS | Cytoplasmic | Negative: Score 0 (no positive cells),  Positive: Score 1 (1-9%), Score 2 (10%-49%), Score 3 (≥50%) | [13] |
| Serotonin | Dako | 5HT-H209/M075801-2 | 1:100 | Ventana^b^ | Mouse, monoclonal | WS | Cytoplasmic | Negative: Score 0 (no positive cells),  Positive: Score 1 (1-9%), Score 2 (10%-49%), Score 3 (≥50%) | [13] |
| SOX10 | Novus Biologicals, Centennial, USA | NBP2-79770 | 1:150 | Leica^a^ | Rabbit, monoclonal | WS | Nuclear | Negative 5 or less SOX10+ sustentacular cells in ≥2 HPFs, Positive if >5 | [21] |
| SSTR2A | Abcam | ab134152 | 1:150 | Leica^a^ | Rabbit, monoclonal | TMA | Membranous | Negative: Score 0 and Score 1+, Positive: Score 2 and Score 3 | [36] |
| SSTR5 | Abcam | UMB4 | 1:2000 | Leica^a^ | Rabbit, monoclonal | TMA | Membranous | Negative: Score 0 and Score 1+, Positive: Score 2 and Score 3 | [36] |
| DLL3 | Abcam | EPR22592-18 | 1:50 | Leica^a^ | Rabbit, monoclonal | TMA | Membranous/ Cytoplasmic | Negative: <1% cytoplasmic and/or membranous positive cells,  low: ≥1%, <50%, high: ≥50% | [37, 38] |

Abbreviations: ASCL1, achaete-scute family bHLH transcription factor 1; OTP, Orthopedia Homeobox; HNF1A, Hepatocyte Nuclear Factor 1 Alpha; ACTH, Adrenocorticotropic Hormone; GRP, Gastrin-releasing peptide; SSTR, Somatostatin Receptor; DLL3, Delta-Like Ligand 3; WS, Whole slide; TMA, Tissue micro array.

Footnote: a) BOND-MAX or BOND-III, Leica Biosystems, Wetzlar, Germany, b) Benchmark XT, Ventana/Roche, Tucson, AZ, USA, c) According to the manufacturer, the antibody is generated against synthetic (human) bombesin coupled to bovine thyroglobulin and cross reacts with gastrin releasing peptide.
