# Supplementary material for: Phenotypic Landscape of Pulmonary Neuroendocrine Tumors: Subtyped by OTP/ASCL1 Expression Correlated with Histology, Hormones and Outcome
Source: Endocr Pathol. 2025 Nov 6;36(1):43. doi: 10.1007/s12022-025-09882-z (PMC12592246; doi:10.1007/s12022-025-09882-z)
Supplement: Supplementary file 7 — (DOCX 21.6 KB) [file 12022_2025_9882_MOESM7_ESM.docx]

Supplementary Table 4: Univariate and multivariate analysis on prognosis free survival in 125 patients with pulmonary neuroendocrine tumor.

|  |  |  | Univariate survival analysis | | | Multivariate survival analysis | | |
| --- | --- | --- | --- | --- | --- | --- | --- | --- |
|  |  | N | Event | Mean survival months | log-rank test *(p-value)* | Hazard Ratio | 95% CI | *p-value* |
| Age | <65 | 57 | 5 | 91 | NS | - | | |
|  | ≥65 | 68 | 9 | 103 |  |  |  |  |
| Sex | Female | 81 | 8 | 58 | NS | - | | |
|  | Male | 44 | 6 | 106 |  |  |  |  |
| Size (cm)^b^ | <2cm | 74 | 7 | 107 | NS | - | | |
|  | ≥2cm | 50 | 7 | 88 |  |  |  |  |
| WHO  Thorax | TC | 96 | 4 | 116 | < 0.0001 | 1 |  | NS |
|  | AC | 29 | 10 | 65 |  | 0.29 | 0.05-1.44 |  |
| WHO  endocrine | G1 | 79 | 3 | 89 | < 0.0001 | 1 |  | 0.04^A^ |
|  | G2 | 43 | 9 | 96 |  | 2.82 | 0.52-15.3 |  |
|  | G3 | 3 | 2 | 16 |  | 21.2 | 1.82-247 |  |
| Nodal status^c^ | pN0 | 88 | 8 | 110 | NS | - | | |
|  | pN1/N2 | 9 | 3 | 46 |  |  |  |  |
| Growth pattern | Trabecular | 35 | 3 | 112 | NS | - | | |
|  | Solid | 90 | 11 | 85 |  |  |  |  |
| Spndle | Absence | 75 | 9 | 107 | NS | - | | |
|  | Presence | 50 | 5 | 57 |  |  |  |  |
| Oncocytic | Absence | 113 | 12 | 107 | NS | - | | |
|  | Presence | 12 | 2 | 87 |  |  |  |  |
| OTP | Negative | 67 | 12 | 99 | 0.006 | - | | NS^B^ |
|  | Positive | 58 | 2 | 23 |  |  |  |  |
| ASCL1 | Negative | 61 | 8 | 106 | NS | - | | |
|  | Positive | 64 | 6 | 58 |  |  |  |  |
| TTF1 | Negative | 77 | 7 | 111 | NS | - | | |
|  | Positive | 48 | 7 | 54 |  |  |  |  |
| CD44^d^ | Negative | 49 | 8 | 83 | NS (0.07) | - | | |
|  | Positive | 25 | 0 | 86 |  |  |  |  |
| SSTR2A^e^ | Negative | 51 | 6 | 50 | NS | - | | |
|  | Positive | 57 | 7 | 108 |  |  |  |  |
| SSTR5^f^ | Negative | 80 | 9 | 86 | NS | - | | |
|  | Positive | 8 | 0 | - |  |  |  |  |
| DLL3^g^ | Negative | 49 | 3 | 93 | NS (0.06) | - | | |
|  | Low expression | 8 | 0 | - |  |  |  |  |
|  | High expression | 32 | 6 | 47 |  |  |  |  |
| Subgroup | O+/A+ | 42 | 2 | 23 | 0.03 | - | | NS^C^ |
|  | O+/A- | 30 | 0 | - |  |  |  |  |
|  | O-/A+ | 22 | 4 | 57 |  |  |  |  |
|  | O-/A- | 31 | 8 | 97 |  |  |  |  |

Abbreviations: TC, Typical carcinoid; AC, Atypical carcinoid; NS, Not significant; CI, Confidence Interval.

Footnote: a) One case with positive resection margin, 14 cases with a follow-up period of less than one month, and 12 cases with unavailable follow-up information were excluded. Data missing in b) 1, c) 28, d) 51, e) 17, f) 37, and g) 36 cases. A) G2 vs G3: Hazard Ratio = 7.52, 95% CI: 1.19-47.6, B) Hazard Ratio not interpretable due to non-convergence, C) O-/A- vs O-/A+: Hazard Ratio = 0.41, 95% CI: 0.11–1.49, other pairwise comparisons not interpretable due to sparse events or convergence issues.
