# Supplementary material for: Phenotypic Landscape of Pulmonary Neuroendocrine Tumors: Subtyped by OTP/ASCL1 Expression Correlated with Histology, Hormones and Outcome
Source: Endocr Pathol. 2025 Nov 6;36(1):43. doi: 10.1007/s12022-025-09882-z (PMC12592246; doi:10.1007/s12022-025-09882-z)
Supplement: Supplementary file 8 — (DOCX 19.9 KB) [file 12022_2025_9882_MOESM8_ESM.docx]

Supplementary Table 5. Comparison of clinicopathologial features of primary resected and metastatic neuroendocrine tumors

|  |  | A. Primary | B. Metastatic | *p* value^A^ |
| --- | --- | --- | --- | --- |
| Total |  | 152 | 21 |  |
| Age | Median (range) | 67 (12-86) | 61 (25-81) | NS |
| Sex | Male | 53 (35%) | 13 (62%) | 0.02 |
|  | Female | 99 (65%) | 8 (38%) |  |
| Ki-67 (%) | Median (range) | 2 (0.2-62) | 9 (1.6-50) | <0.0001^C^ |
| Growth pattern | Solid | 113 (74%) | 18 (86%) | NS |
|  | Trabecular | 39 (26%) | 3 (14%) |  |
| Cellular features | Spindle | 64 (42%) | 13 (62%) | NS |
|  | Oncocytic | 14 (9%) | 0 |  |
|  | Other | 74 (49%) | 8 (38%) |  |
| GRP^a^ | Negative | 41 (32%) | 6 (30%) | NS |
|  | Positive | 89 (68%) | 14 (70%) |  |
| ACTH^b^ | Negative | 87 (57%) | 18 (90%) | 0.005 |
|  | Positive | 65 (43%) | 2 (10%) |  |
| Calcitonin^c^ | Negative | 122 (80%) | 16 (80%) | NS |
|  | Positive | 30 (20%) | 4 (20%) |  |
| Serotonin^d^ | Negative | 131 (86%) | 15 (79%) | NS |
|  | Positive | 21 (14%) | 4 (21%) |  |
| Subtypes | OTP+/ASCL1+ | 58 (38%) | 7 (33%) | NS |
|  | OTP+/ASCL1- | 35 (23%) | 3 (14%) |  |
|  | OTP-/ASCL1+ | 22 (14%) | 7 (33%) |  |
|  | OTP-/ASCL1- | 37 (24%) | 4 (19%) |  |
| OTP | Negative | 59 (39%) | 11 (52%) | NS |
|  | Positive | 93 (61%) | 10 (48%) |  |
| ASCL1 | Negative | 72 (47%) | 7 (33%) | NS |
|  | Positive | 80 (53%) | 14 (67%) |  |
| TTF1^e^ | Negative | 88 (58%) | 6 (32%) | 0.03 |
|  | Positive | 64 (42%) | 13 (68%) |  |
| HNF1A^f^ | Negative | 69 (70%) | 15 (83%) | NS |
|  | Positive | 30 (30%) | 3 (17%) |  |
| CD44^g^ | Negative | 60 (63%) | 11 (65%) | NS |
|  | Positive | 36 (37%) | 6 (35%) |  |
| Sustentacular cells^h^ | Absence | 83 (58%) | 9 (60%) | NS |
|  | Presence | 61 (42%) | 6 (40%) |  |
| SSTR2A^i^ | Negative | 61 (46%) | 9 (47%) | NS |
|  | Positive | 73 (54%) | 10 (53%) |  |
| SSTR5^j^ | Negative | 103 (90%) | 14 (78%) | NS |
|  | Positive | 11 (10%) | 4 (22%) |  |
| DLL3^k^ | Negative | 62 (53%) | 7 (37%) | NS |
|  | Low expression | 11 (9%) | 2 (11%) |  |
|  | High expression | 43 (37%) | 10 (53%) |  |

Abbreviations: NS, not significant

Footnote: Data missing in a) 1 in B, b) 1 in B, c) 1 in B, d) 2 in B, e) 2 in B, f) 56 (53 in A, 3 in B), g) 60 (56 in A, 4 in B), h) 14 (8 in A, 6 in B), i) 19 (18 in A, 1 in B), j) 41 (38 in A, 3 in B), k) 38 (36 in A, 2 in B), A: Pearson´s chis-quare test among five subtypes, B: Kruskal-Wallis Test.
